# A naturally occurring variant of SHLP2 is a protective factor in Parkinson’s disease

**DOI:** 10.1038/s41380-023-02344-0

**Published:** 2024-01-03

**Authors:** Su-Jeong Kim, Brendan Miller, Nicolas G. Hartel, Ricardo Ramirez, Regina Gonzalez Braniff, Naphada Leelaprachakul, Amy Huang, Yuzhu Wang, Thalida Em Arpawong, Eileen M. Crimmins, Penglong Wang, Xianbang Sun, Chunyu Liu, Daniel Levy, Kelvin Yen, Giselle M. Petzinger, Nicholas A. Graham, Michael W. Jakowec, Pinchas Cohen

**Affiliations:** 1 The Leonard Davis School of Gerontology, University of Southern California, Los Angeles, CA USA; 2 Mork Family Department of Chemical Engineering and Materials Science, University of Southern California, Los Angeles, CA USA; 3 Environmental Toxicology Program, Chulabhorn Graduate Institute, Bangkok, 10210 Thailand; 4 The Population Sciences Branch, National Heart, Lung, and Blood Institute, National Institutes of Health, Bethesda, MD USA; 5 Department of Biostatistics, Boston University School of Public Health, Boston, MA USA; 6 Boston University’s and National Heart, Lung, and Blood Institute’s Framingham Heart Study, Framingham, MA USA; 7 Department of Neurology, University of Southern California, Los Angeles, CA USA; 8 Norris Comprehensive Cancer Center, University of Southern California, Los Angeles, CA USA; 9 Department of Biokinesiology and Physical Therapy, The George and MaryLou Boone Center for Parkinson’s Disease Research, University of Southern California, Los Angeles, CA USA

**Keywords:** Cell biology, Neuroscience

## Abstract

Mitochondrial DNA single nucleotide polymorphisms (mtSNPs) have been associated with a reduced risk of developing Parkinson’s disease (PD), yet the underlying mechanisms remain elusive. In this study, we investigate the functional role of a PD-associated mtSNP that impacts the mitochondrial-derived peptide (MDP) Small Humanin-like Peptide 2 (SHLP2). We identify m.2158 T > C, a mtSNP associated with reduced PD risk, within the small open reading frame encoding SHLP2. This mtSNP results in an alternative form of SHLP2 (lysine 4 replaced with arginine; K4R). Using targeted mass spectrometry, we detect specific tryptic fragments of SHLP2 in neuronal cells and demonstrate its binding to mitochondrial complex 1. Notably, we observe that the K4R variant, associated with reduced PD risk, exhibits increased stability compared to WT SHLP2. Additionally, both WT and K4R SHLP2 show enhanced protection against mitochondrial dysfunction in in vitro experiments and confer protection against a PD-inducing toxin, a mitochondrial complex 1 inhibitor, in a mouse model. This study sheds light on the functional consequences of the m.2158 T > C mtSNP on SHLP2 and provides insights into the potential mechanisms by which this mtSNP may reduce the risk of PD.

## Introduction

Mitochondria are implicated in the pathogenesis of idiopathic Parkinson’s disease (PD) [1]. A defect of mitochondrial complex I has been observed in the substantia nigra dopaminergic neurons of postmortem PD brains [2]. Recent study showed disruption of mitochondrial complex 1 induces progressive parkinsonism [2]. The complex I inhibitor MPTP (N-methyl-4-phenyl-1,2,3,6-tetrahydropyridine) causes parkinsonism and nigrostriatal dopaminergic degeneration in humans and animal models [3, 4]. Several monogenic forms of PD are caused by mutations in genes coding for mitochondrial function. For instance, the second most common cause of autosomal recessive early-onset PD can be attributed to mutations in the PINK1 gene, a mitochondrial serine/threonine kinase [1]. PINK1 plays a significant role in maintaining mitochondrial homeostasis, enhancing mitochondrial fission, and promoting mitophagy [1]. Conversely, the loss of PINK1 leads to a wide range of mitochondrial dysfunction [1]. Moreover, genetic studies of large cohorts of ethnically homogeneous subjects revealed that mitochondrial DNA (mtDNA) polymorphisms play a role in the risk of developing PD [5–8]. Although a strong association has been found by population genetics, the functional effects of the mtDNA polymorphisms are underexplored.

MDPs are bioactive microproteins that are encoded from small open reading frames in mitochondrial DNA. Since the first MDP named humanin was discovered in 2001, eight more microproteins have been identified that play essential roles in pathophysiology of age-related diseases [9]. The levels of some MDPs decline with age in mice and humans, and the administration of MDPs can exert beneficial effects in in vitro and in vivo models of age-related disease, including Alzheimer’s disease, cardiovascular disease, and type 2 diabetes (T2D) [10, 11]. One of the MDPs, a small humanin‐like peptide 2 (SHLP2), is encoded from the non-coding RNA ASncmtRNA-1/2 in the mtDNA and plays an essential role in various cellular processes [12]. SHLP2 improves mitochondrial metabolism by increasing respiration and biogenesis, reducing reactive oxygen species, and decreasing mtDNA oxidation [12]. SHLP2 has also been suggested to be a possible treatment for age-related macular degeneration (AMD). Work from the Kenney lab has found that SHLP2 administration was able to prevent a number of negative effects in an in vitro AMD model [13]. The misfolding and subsequent aggregation of Islet amyloid polypeptide (IAPP) induces beta cell apoptosis, oxidative damage, and mitochondrial dysfunction, playing critical roles in the pathogenesis of type 2 diabetes (T2D). SHLP2 acts as a chaperone and directly binds to the misfolded, seeding-capable IAPP species and blocks amyloid seeding [14]. The chaperone-like activity can link the anti-apoptotic roles and beneficial metabolic effect of SHLP2 against IAPP aggregation, suggesting that SHLP2 has potential as a T2D therapeutic. Circulating levels of SHLP2 have also been correlated with android and liver fat and inversely correlated with prostate cancer risk [15, 16].

Recent studies show that mitochondrial DNA single nucleotide polymorphisms (mtSNPs) within coding regions of MDPs are associated with age-related deficits and could help discover novel microproteins. For example, mtSNP m.2706 A > G in humanin predicts lower circulating humanin levels and worse cognitive decline [17]. Administration of humanin in old mice prevents age-related cognitive decline [17]. The m.12372 G > A is associated with Alzheimer’s disease (AD) and revealed a novel MDP called SHMOOSE [11]. Additionally, m.1382 A > C – a mtSNP that changes the 14th amino acid of the wild-type MDP MOTS-c from the amino acid lysine (K) to glutamine (Q) – predisposes individuals of East Asian decent to Type 2 Diabetes [18]. Importantly, K14Q MOTS-c is less effective as a metabolic regulator in cell and animal models and is associated with suppressed insulin-sensitizing effects and higher body fat levels [18].

The mtSNP m.2158 T > C is associated with reduced risk of PD [6]. However, the underlying biological mechanisms responsible for this effect remains a gap in our knowledge. Here, we present a series of experiments that explore the effects of the m.2158 T > C allelic form of SHLP2 (termed K4R SHLP2) on mitochondrial function. Elucidating mechanisms by which SHLP2 and the K4R variant impact mitochondrial function may guide novel therapeutic modalities focused on restoring mitochondrial function in PD.

## Materials and methods

### Human cohorts

#### Health Retirement Study (HRS)

##### Participants

Data are from the Health and Retirement Study (HRS), a nationally representative sample of older Americans over age 50 [19, 20] in the contiguous United States. The present analysis was limited to participants who self-reported their race as white/Caucasian, verified by principal components analysis of ancestry markers, in order to assess effects of the SHLP2 minor allele only found in European ancestry groups. The analytical sample for the HRS included individuals who had available genetic data, at least one measure of total cholesterol data (assessed in 2006, 2010, 2012, or 2014) and relevant covariate data (*N* = 9,774), with a sub-sample available for the analysis with LDL (assessed in 2016; *N* = 4,297). The HRS data collection is supported by: U01 AG009740 to the University of Michigan.

##### SHLP2 Mitochondrial Single Nucleotide Polymorphism (mtSNP)

For HRS, genotype data were accessed from the National Center for Biotechnology Information Genotypes and Phenotypes Database (dbGaP [21]). Genotyping was conducted on over 15,000 individuals using either the Illumina HumanOmni2.5-4v1 (2006 and 2008) and HumanOmni2.5-8v1 (2010) arrays and was performed by the NIH Center for Inherited Disease Research (CIDR). Standard quality control procedures were implemented by the University of Washington Genetic Coordinating Center [22]. Further detail is provided in the HRS documentation [23]. The SHLP2 allele coding (binary coded as 0 or 1 for a mitochondrial SNP) was extracted using PLINK 1.9 [24, 25]. Cholesterol Biomarkers: The HRS collected blood-based biomarkers including total cholesterol and high-density lipoprotein (HDL) levels on half of the sample in 2006, and the other half in 2008, with additional individuals captured in the 2010 or 2012 data collection waves. Detailed information on collection and assay are provided elsewhere [26, 27]. Total cholesterol values were adjusted for medication use as done previously in the FHS study. For individuals reporting taking medications to lower their cholesterol, levels were divided by .80 to produce an adjusted total cholesterol value [28]. In 2016, venous blood samples were collected on a subsample in HRS to assay and calculate low density lipoprotein (LDL) levels with detailed information provided previously [29]. Covariates: In HRS, covariates included age at biomarker assessment, gender (0=female, 1=male), and five principal components to reduce type 1 error due to differences in underlying population substructure [30, 31]. Detailed descriptions of the processes employed for running principal components analysis, including SNP selection, are provided by HRS [23], and follow gold standard methods [32]. Models constructed for HDL and LDL were adjusted for medication use (0=no, 1=yes), if respondents reported using medication to lower their cholesterol.

##### Statistical analysis

In HRS, multivariable linear regression models were run to test for the association between SHLP2 and dependent variables of total cholesterol (medication adjusted value), HDL and LDL, adjusting for age, gender, principal components, and medication use (for HDL and LDL) using SAS 9.4.

#### Cardiovascular Health Study (CHS) and Framingham Heart Study (FHS)

##### Participants: The Cardiovascular Health Study (CHS) (*n* = 2,772)

The Cardiovascular Health Study (CHS) originated in 1988 to recruit participants from four U.S. communities [33]. The CHS has been followed since then to study coronary heart disease and stroke in 5,888 elderly adults aged 65 years and older. The first exam began in June 1989. A second comprehensive exam began 3 years after the first exam. All study participants provided written informed consent for genetic studies. A total of *n* = 2,772 CHS participants of white/Caucasian (mean age 74 and 57% women) with whole genome sequencing were included in this study. Cohort acknowledgement/support: The CHS (phs001368.v1.p1) was supported by contracts 75N92021D00006, HHSN268201200036C, HHSN268200800007C, HHSN268201800001C, N01HC55222, N01HC85079, N01HC85080, N01HC85081, N01HC85082, N01HC85083, N01HC85086, and grants U01HL080295, R01HL105756, and U01HL130114 from the NHLBI, with additional contribution from the National Institute of Neurological Disorders and Stroke (NINDS). Additional support was provided by R01AG023629 from the National Institute on Aging (NIA). A full list of principal CHS investigators and institutions can be found at CHS-NHLBI.org. Sequencing was supported and conducted in collaboration with Baylor University (HHSN268201600033I, 3U54HG003273-12S2, HHSN268201500015C) contracts from NHLBI.

##### The Framingham Heart Study (FHS) (*n* = 3,621)

The FHS is a single-site, community-based, prospective study that was initiated in 1948 to investigate the risk factors for CVD [34]. The second generation [35] was recruited in 1971 and the third generation [36] was recruited between 2002 and 2005. The first generation has been examined every 2 years. The second generation has been examined every 4–8 years. The third generation has had three examinations. A small number of spouse individuals of the second generation was examined at the same time when the third generation had their first examination. A total of 4,196 FHS participants were whole genome sequenced by TOPMed; of those, 376 were the first generation, 2218 were the second generation and 95 were spouses of the second generation participants; and 1507 were the third generation participants. All study participants provided written informed consent for genetic studies. This study included 3,621 FHS participants. The WGS for FHS (phs000974) was performed at the Broad Institute of MIT and Harvard (3R01HL092577-06S1 and 3U54HG003067-12S2). The FHS acknowledges the support of contracts NO1-HC-25195, HHSN268201500001I and 75N92019D00031 from the National Heart, Lung and Blood Institute and grant supplement R01 HL092577-06S1 for this research. We also acknowledge the dedication of the FHS study participants without whom this research would not be possible. Dr. Vasan is supported in part by the Evans Medical Foundation and the Jay and Louis Coffman Endowment from the Department of Medicine, Boston University School of Medicine. X.L., S.S., C.L.S, and C.L. are also supported by R01AG059727. C.L.S and S.S are also supported by AG052409, AG054076 and AG059421.

##### Cholesterol biomarkers

At each of the health exams, the FHS collected blood-based biomarkers including total cholesterol, high-density lipoprotein (HDL), and triglyceride. We matched these lipid measurements to the time when blood was drawn for whole genome sequencing LDL (mg/dL) was calculated as (TC - HDL - TRIG/5) in individuals with TRIG < 400 mg/dL TC values [37].

##### Covariates

Covariates included age at whole genome sequencing, gender (0=female, 1=male), and five principal components to reduce type 1 error due to differences in underlying population substructure [30, 31]. In TOPMed, principle components calculated PCA using PC-AiR [38]. Models constructed for HDL and LDL were adjusted for medication use (0=no, 1=yes), if respondents reported using medication to lower their cholesterol.

##### mtDNA homoplasmy derivation in FHS and CHS

Whole blood derived DNA was used for WGS from TOPMed sequencing centers. In analyzing sequencing data, the coverage was defined as the number of reads that were mapped to a given nucleotide in the reconstructed sequence. The average coverage was ~39x across samples in TOPMed. The program *fastMitoCalc* of the software package *mitoAnalyzer* [39] was used to identify mtDNA homoplasmy based on derived alternative allele fractions (AAFs) after comparing sequencing reads to rCRS [40]. We set an mtDNA locus as missing if the coverage was <250-fold [39]. We compared homoplasmic alleles called by previous genotyping arrays to the ones derived from TOPMed whole genome sequencing in the same individuals [41]. Individuals with >two inconsistent homoplasmic alleles (out of ~200 mtDNA variants genotyped by the arrays) were red-flagged and discrepancies examined. We also compared the homoplasmic variants within maternal lineage members. We also removed several sites listed in ‘blacklisted sites’ (301,302,310, 316, 3107, and 16182 mtDNA loci) recommended by GATK (https://console.cloud.google.com/storage/browser/gatk-best-practices/mitochondria-pipeline/ We determined that the >97% AAF was used to determine homoplasmy based on the calling results from 3 samples that each had 4 repeated whole genome sequencing data.

##### Statistical analysis

In FHS and CHS, multivariable linear regression models were performed to test for the association between SHLP2 and dependent variables of total cholesterol (medication adjusted value), HDL and LDL, adjusting for age, gender, five principal components, and medication use (for HDL and LDL) using R software version 4.0.0.

#### Mass spectrometry of SHLP2 identification

SH-SY5Y cells were differentiated using 10 μM of retinoic acid, as previously described [42]. Approximately 8 × 10^7^ differentiated cells were washed once with ice cold PBS and immediately lysed using 50 mM HCl, 2 mM TCEP, 0.05% Triton X-100 (lysis buffer) at room temperature for 15 minutes. This lysis buffer has previously yielded high yield of microproteins from cell extracts [43]. After the 15-minute lysis duration, the sample was centrifuged at 25,000 x g for 30 minutes at 4°C. Cleared lysate was then passed through a 5 μm strainer (pluriSelect Life Science, Leipzig, Germany). A BCA protein assay (Thermo Scientific, Waltham, MA) was used to quantify protein concentration. Synthetic light (MGVKFFTLSTRFFPSVQRAVPLWTNS) and heavy (MGVKFFTLSTRFFPSVQ{Arg(13C6 15N4)}AVPLWTNS) versions sequence of SHLP2 were combined and reduced with dithiothreitol, alkylated with iodoacetamide, digested with trypsin and STAGE tip desalted on a C8 core. Digests were loaded onto a nanoscale UHPLC system (EASY-nLC1200, Thermo Scientific) connected to a Q Exactive Plus hybrid quadrupole-Orbitrap mass spectrometer equipped with a nanoelectrospray source (Thermo Scientific). Peptides were separated by a reversed-phase analytical column (PepMap RSLC C18, 2 μm, 100 Å, 75 μm × 25 cm) (Thermo Scientific). Flow was set to 200nL/min starting with 3% buffer B (0.1% formic acid, 80% acetonitrile) to 38%B in 110 minutes, then washed with 75%B for 1 minute and then 85%B for 10 minutes and held at 85%B for 9 minutes. Column temperature was set to 55 C. Spray voltage was set to 2000V and all experiments were ran in positive mode. A series of injections ranging from 700 pg – 500 ng of protein were performed on a data-dependent acquisition (DDA) method and data imported to Skyline where a parallel reaction monitoring (PRM) inclusion list was generated for the most common peptides identified from both the light and heavy SHLP2. The DDA method full scan range was from 400-900 m/z with a 70,000 resolution and maximum injection time of 120 ms with 1e6 AGC target, and 17,500 resolution for ddMS2 scans with a maximum injection time of 120 ms and 1e5 AGC target. Isolation width was set to 1.0 m/z with a normalized collision energy of 26. Dynamic exclusion was enabled and set to 20 seconds. The PRM method was run at 35,000 resolution with a 700 ms injection time and 5e5 AGC target with the same isolation width and collision energy as the DDA method. Lysate from SH-SY5Y cells were enriched by C8 column enrichment, concentrated by methanol/ chloroform precipitation, reduced, alkylated, digested with trypsin, desalted with C8 STAGE tip and run in PRM mode with the inclusion list. A parallel procedure was done with the heavy labeled version of SHLP2 spiked into SH-SY5Y lysate. RAW files were searched both in Skyline and Proteome Discoverer 2.2 using SEQUEST and MS AMANDA 2.0 using a mitochondrial protein FASTA file containing SHLP2 sequence. Cleavage was set to trypsin allowing for up to two missed cleavages, with oxidation of methionine (+15.995 Da), heavy arginine (+10.008 Da), and acetylation of protein N-terminus (+42.011 Da) set as variable modifications. Carbamidomethylation of cysteine (+57.021 Da) was set as a static modification. Precursor mass tolerance was set to 10 ppm and fragment ion tolerance was set to 0.02 Da. Validation was performed by Fixed Value PSM Validator node in Proteome Discoverer with maximum delta Cn set to 0.05. Identified peptides were further confirmed by checking exact m/z values and retention times to those observed in the synthetic protein experiments.

#### Reagents, plasmids, antibodies

SHLP2 (small humanin-like peptide 2) peptides were synthesized by Genscript (Piscataway, NJ, USA). Peptides were initially dissolved in Milli-Q water. Cycloheximide (Sigma, St. Louis, MO, USA) was used for pulse chase experiment in culture. pcDNA3.1-WT SHLP2-EGFP and pcDNA3.1-K4R SHLP2 -EGFP were cloned in Genscript (Piscataway, NJ, USA). Transient transfection was performed with Lipofectamine 3000 as recommended by the supplier (ThermoFisher Scientific). After transfection, the cells were incubated for 5 h, switched into a complete culture medium, and further incubated for an additional 36 h. The following antibodies were used in this study: GFP (1:1000), Lamin B1 (1:1000), S6K (1:1000), anti-rabbit IgG, HRP-linked antibody (1:30000), and anti-mouse IgG, HRP-linked antibody (1:30000). (all Cell Signaling Technology), ACTIN (1:2000) and GRSF-1 (1:1000; sigma), Tom20 (1:100; Santa Cruz Biotechnology). Customized rabbit anti-SHLP2 antibody was produced by Harlan Laboratories (Indianapolis, IN, USA).

#### Cell culture and treatment

HEK293 were purchased from ATCC (Manassas, VA, USA) and HeLa expressing HA- EGFP- OMP25 (HA-MITO) or myc-EGFP-OMP25 (CON-MITO) cells were gifts from Dr. Sabatini [44]. The HEK293 and HeLa cells were cultured in DMEM + 10% FBS (FBS; ThermoFisher Scientific) at 37°C in 5% CO2. Cycloheximide were treated into HEK293 cells for the indicated time. Wild type and TFAM heterozygous mouse embryonic fibroblasts cells were Cultured in DMEM + 15% FBS at 37°C in 5% CO2.

#### Western blot analysis

Cells were lysed with RIPA Lysis and Extraction Buffer (ThermoFisher Scientific) plus the Halt protease & phosphatase inhibitor cocktail (ThermoFisher Scientific). The lysates were incubated on ice for 10 min then homogenized using a sonicator, and the supernatant was collected by centrifugation at 15,000 x g for 15 min at 4 °C. Protein content in the cellular lysates was quantified using the Pierce™ BCA Protein Assay Kit (ThermoFisher Scientific). Predetermined amounts of proteins (10-30μg) were separated on 8-16% SDS-PAGE gels and blotted onto PVDF membranes (Biorad, Hercules, CA, USA). Membranes were incubated with primary antibody at 4 °C overnight according to the manufacturer’s instructions. After several washes with Tris-buffered saline containing 0.1% Tween-20, membranes were incubated at room temperature for 1 hr with the appropriate HRP-conjugated secondary antibody. Clarity™ Western ECL substrate (Biorad) was used for detecting specific bands. Membranes were imaged on a Bio-Rad ChemiDoc XRS^+^ imager. If necessary, relative intensities of the bands in each condition were measured using Image J, a free software program provided by National Institute of Health (Bethesda, Maryland, USA).

#### Mitochondria isolation using HeLa expressing HA- EGFP- OMP25 (HA-MITO)

Mitochondria isolation from HeLa cells expressing HA-EGFP-OMP25 (HA-MITO) or myc-EGFP-OMP25 (CON-MITO) was performed according to the published article [44]. Briefly, cells were lysed in KPBS buffer (136 mM KCl, 10 mM KH2PO4, pH 7.25) and homogenized with 25 strokes of a 2 ml homogenizer. The homogenate was spun down at 1000 g for 2 min. The supernatant was incubated with anti-HA magnetic beads on an end-over-end rotator for 3.5 min. The beads were washed with KPBS buffer, incubated for 10 min with lysis buffer (1% triton X-100 lysis buffer with protease inhibitor cocktail), and spun down at 17000 g for 10 min. The supernatants were used for western blot.

#### Mitochondria and cytoplasmic fractionation

Mitochondrial and cytoplasmic fractionation from HEK293 cells was performed using the Qproteome Mitochondrial Isolation kit (Qiagen, Valencia, CA, USA) according to the manufacturer’s instruction. Briefly, cells were resuspended in Lysis Buffer and incubated for 10 min. Cell lysates were centrifuged at 1,000 x g for 10 min at 4 °C. The supernatant was collected for the cytosolic fraction. The pellet was resuspended in Disruption Buffer. After homogenization, resuspension was centrifuged at 1,000 x g for 10 min at 4 °C. The supernatant was collected and centrifuged at 6,000 x g for 10 min. The pellet contains mitochondria.

#### Sodium carbonate extraction

Mitochondrial extraction from HEK293 cells was perform using the Qproteome Mitochondrial Isolation kit (Qiagen) according to the manufacturer’s instruction. Mitochondria pellets isolated from HEK293 cells were resuspended in buffer (320 mM Sucrose, 1 mM EDTA, 10 mM Tris-Cl pH 7.4) containing either 1% Triton X or 0.1 M Na_2_CO_3_ pH 11. The resuspensions were incubated at 4  °C for 1 h with gentle nutation. Supernatant and insoluble pellet were separated by centrifugation at 20,000 x g for 30 min, and the supernatant was gently removed without disturbing the pellet. Equal fractions of supernatant and pellet were analyzed by SDS-PAGE immunoblotting.

#### Immunocytochemistry

HEK293 cells cultured on coverslips were transiently transfected with WT or K4R SHLP2-EGFP for 36 hr and were cultured on coverslips and then fixed with 4% paraformaldehyde for 10 min at room temperature. After fixation, the cells were permeabilized with 0.2% Triton X-100 in phosphate-buffered saline (PBS) for 10 minutes at room temperature and were blocked in PBS containing 0.2% Triton X-100 and 1% bovine serum albumin (BSA) for 1 hour at room temperature. Cells were then incubated with mouse anti-Tom20 antibody (1:100; Santa Cruz Biotechnology) in PBS containing 0.2% Triton X-100 and 1% BSA at 4 °C overnight. After three washes with PBS, the cells were further incubated with Alexa Fluor 568-conjugated donkey anti-mouse IgG (1:200; Invitrogen) in PBS containing 0.2% Triton X-100 and 1% BSA for 1 hour at room temperature in dark. Nuclei were stained for 5 minutes at room temperature in PBS containing Hoechst 33258 (2 mg/ml; Invitrogen). Coverslips were mounted with ProLong Gold antifade reagent (Invitrogen). Images were acquired with a Keyence microscope (Keyence corporation of America, Itasca, IL). For immunostaining of COX IV antibody, fixed and permeabilized cells were incubated with anti-COX IV antibody (1:200; abcam) and Alexa Fluor 448-conjugated donkey anti-rabbit IgG (1:200; Invitrogen).

#### RNA extraction and qRT-PCR

Total RNA was extracted from cells and tissues using the Direct-zol RNA MiniPrep Plus (ZYMO RESEARCH, Irvine, CA, USA). The protocol was performed according to manufacturer’s instructions and RNA was measured at 260 nm with the Nanodrop system (ThermoFisher Scientific, Wilmington, DE, USA). RNA purity and quality were evaluated by 260/280 and 260/230 ratios. 1 mg of RNA was used for reverse transcription by using SuperScript IV Reverse Transcriptase and oligo dT 20 primers (ThermoFisher Scientific) according to the manufacturer’s instruction. For quantitative real-time PCR (CFX system, Biorad) Ssoadvanced Universal SYBR green supermix (Biorad) was used to amplify cDNA and quantify the relative gene expression analysis. The 2^−ΔΔCT^ method is used for relative gene expression analysis. This method shows the fold increase (or decrease) of the target gene in the test sample relative to the calibrator sample and is normalized to the expression of a reference gene. First, we normalized the C_T_ of the target gene to that of the reference gene for both the test and calibrator sample (ΔC_T_). Secondly, we normalized the ΔC_T_ of the test sample to the ΔC_T_ of the calibrator sample (ΔΔC_T_). Finally, we calculated the expression ratio, 2^−ΔΔCT^. RPS13 was used as refence gene. The primers used for amplification are: GFP Forward primer (5’ GAACCGCATCGAGCTGAA 3’) and Reverse primer (5’ TGCTTGTCGGCCATGATATAG 3’) and RPS13 Forward primer (5′CATGGCTCGCTC GGTGAC3′), and Reverse primer (5′CAGTTCAGTATGTTCGGCTTCC3′).

#### Pulse Chase experiment

Cycloheximide, an inhibitor of protein biosynthesis, was used to examine protein stability in cells. HEK293 cells were seeded in 6-well plate. WT and K4R SHLP2 were transfected into HEK293 cells. After 24 hr, 100ug/ml cycloheximide was treated for the indicated time. The protein quantity from western blot was examined with Image J.

#### DNA extraction and mitochondrial DNA copy number measurement

Genomic DNA from 300,000 cells was extracted with the DNeasy Blood & Tissue Kits (Qiagen). The mitochondrial copy number was estimated by real-time PCR (CFX Connect Real-Time System, Biorad) using two mtDNA targets (ND1, CYB) and two nuclear DNA targets (ApoB, B2M) (IDT, CA, USA). Real-time PCR was performed by using SsoAdvancedTM Universal SYBR® Green Supermix, following the protocol provided by the manufacturer. The ratio of mtDNA to nuclear DNA was calculated by averaging the differences in amplification efficiency between ND1 and ApoB (∆Ct) and between CYB and B2M (∆Ct). The primers used for amplification are: ND1 Forward primer (5’-gccgtagcccaaacaatttc-3’) and Reverse primer (5’-caggctggcagaagtaatcata-3’); CYB Forward primer (5’-aggagacccagacaactaca-3’) and Reverse primer (5’-tgagcgtagaatggcgtatg-3’); ApoB Forward primer (5’-cgtgggctccagcattcta-3’) and Reverse primer (5’-tcaccagtcatttctgcctttg-3’); B2M Forward primer (5’-atgggaagccgaacatactg-3’) and Reverse primer (5’-cagtctcagtgggggtgaat-3’).

#### MTT assay

The viability of cells after SHLP2 treatment were determined using the tetrazolium dye MTT [3-(4,5-dimethylthiazol-2-yl)-2,5-diphenyltetrazolium bromide] assay. All the treatments were done using 5,000 cells in 96 well plate. The purple insoluble formazan was read on a microplate reader at a wavelength of 570 nm.

#### Biotin-Phenol labeling in live cells and western blot of biotin-phenol labeling

Biotin-phenol labeling in live cells was performed according to previously published protocol [45, 46]. Briefly, the plasmid harboring SHLP2-APEX fusion proteins (SHLP2-APEX-myc) or the APEX control (Flag-APEX-myc) were transiently transfected into HEK293 cells using Lipofectamine 3000. Twenty-four hours post-transfection, the cell culture medium was changed to fresh growth medium containing 500μM biotin-tyramide (CDX-B0270, Adipogen). After incubation at 37 °C for 30 min, H_2_O_2_ was added to each plate at a final concentration of 1 mM, and the plates were gently agitated for 1 min. Cells were then washed three times with a quenching solution [5 mM Trolox, 10 mM sodium azide, and 10 mM sodium ascorbate in PBS], and the pellet was collected by centrifugation at 1000 g for 5 min. Cell pellets were lysed on ice for 20 min in RIPA buffer supplemented with a protease/phosphatase inhibitor and 1 mM phenylmethanesulfonyl fluoride (PMSF) followed by centrifugation at 20,000 g for 20 min at 4 °C to remove cell debris. Cell lysates were added to prewashed streptavidin agarose resin, rotated at 4 °C for 4 hr, and then washed three times. Bound proteins were eluted with 2X SDS loading buffer and analyzed by Western blotting.

#### SHLP2 peptide column

Isolated mitochondrial protein was prepared from HEK293 cells. The cells were scraped and homogenized in mitochondrial isolation buffer (20 mM potassium HEPES, pH7.4, 75 mM Sucrose, 10 mM EDTA, 215 mM Mannitol), followed by differential centrifugation using 700 g for 10 min and 10,500 g for 10 min, respectively. The mitochondrial pellets were lysed using 0.1% NP-40 lysis buffer (50 mM Tris-chloride, pH 8.0, 150 mM NaCl, 0.1% Nonidet P-40 Substitute 11332473001, Roche) with Halt™ Protease Inhibitor Cocktail (ThermoFisher). The mitochondrial lysate concentrations were measured using BCA method (ThermoFisher). The scrambled peptide and SHLP2 peptide were pre-immobilized on agarose beads from CarboxyLinkTM kit (ThermoFisher). 1.6-1.9 mg of mitochondrial protein extract were incubated in the equilibrated column at 4 °C overnight on a rotator. The columns were washed, then the bound proteins were eluted by Elution Buffer according to the manufacturer’s protocol. The 280 nm absorbance of each elution was measured to observe protein concentrations. The eluted proteins were concentrated using Amicon® Ultra-15 Centrifugal Filter Devices (Millipore) before being analyzed by mass spectrometry.

#### Biotin-Phenol labeling mass spectrometry and SHLP2 peptide column mass spectrometry

Biotin-Phenol labeling mass spectrometry- Digestion and Desalting: Proteins bound to streptavidin beads were reduced and alkylated via sequential 20 min incubations of 5 mM TCEP and 10 mM iodoacetamide at room temperature in the dark while being mixed at 1200 rpm in an Eppendorf thermomixer. Proteins were then digested by the addition of 0.1 μg Lys-C (Promega) and 0.8 μg Trypsin (Thermo Scientific, 90057) while shaking 37 °C overnight. The digested samples were quenched by addition of formic acid to a final concentration of 5% (v/v). Each sample was desalted through C18 tips (Thermo Scientific, 87784) and then resuspended in 5% formic acid before being analyzed by LC-MS/MS.

##### SHLP2 peptide column mass spectrometry-Digestion and Desalting

SHLP2 peptide column elution samples were mixed with same volume of digestion buffer (8 M Urea, 0.1 M Tris-HCl pH 8.5), then each sample was reduced and alkylated via sequential 20-minute incubations with 5 mM TCEP and 10 mM iodoacetamide at room temperature in the dark while being mixed at 1200 rpm in an Eppendorf thermomixer. 18 μl of carboxylate-modified magnetic beads (CMMB and also widely known as SP3) was added to each sample. Ethanol was added to a concentration of 50% to induce protein binding to CMMB. CMMB were washed 3 times with 80% ethanol and then resuspended with 50 μl 50 mM TEAB. The protein was digested overnight with 0.1 μg LysC (Promega) and 0.8 μg trypsin (Thermo Scientific, 90057) at 37  °C. Following digestion, 1 ml of 100% acetonitrile was added to each to sample to increase the final acetonitrile concentration to over 95% to induce peptide binding to CMMB. CMMB were then washed 3 times with 100% acetonitrile and the peptide was eluted with 50 μl of 2% DMSO. Eluted peptide samples were dried by vacuum centrifugation and reconstituted in 5% formic acid before analysis by LC-MS/MS.

##### LC-MS Acquisition and analysis

Peptide samples were separated on a 75 μM ID, 25 cm C18 column packed with 1.9 μM C18 particles (Dr. Maisch GmbH) using a 140-minute gradient of increasing acetonitrile concentration and injected into a Thermo Orbitrap-Fusion Lumos Tribrid mass spectrometer. MS/MS spectra were acquired using Data Dependent Acquisition (DDA) mode. MS/MS database searching was performed using MaxQuant (1.6.10.43) against the human reference proteome from EMBL (UP000005640_9606 HUMAN Homo sapiens, 20874 entries). Statistical analysis of MaxQuant label-free quantitation data was performed with the artMS Bioconductor package which performs the relative quantification of protein abundance using the MSstats Bioconductor package (default parameters). The abundance of proteins missing from one condition but found in more than 2 biological replicates of the other condition for any given comparison were estimated by imputing intensity values from the lowest observed MS1-intensity across samples and *p* values were randomly assigned to those between 0.05 and 0.01 for illustration purposes.

#### Co-expression network analysis

The SHLP2 transcript was correlated to all gene transcripts using data from iPSCs-derived dopaminergic neurons from the Cedars-Sinai Board of Governors Regenerative Medicine Institute. Briefly, counts from RNASeq data were produced by SNAPR and normalized using edgeR-based implementation of the trimmed mean of M-values (TMM), which calculated counts per million (CPM). These normalized counts were assigned to the gene levels. Since the SHLP2 ORF was not annotated, we re-analyzed available BAM files and counted transcripts, using the summarizeOverlaps method as part of the Bioconductor package in R, that mapped back to the humanin ORF against a custom mitochondrial ORFome database. We then extracted normalized humanin transcript counts and conducted a Pearson correlation for the SHLP2 transcript against normalized counts of all genes, correcting for multiple hypothesis testing using a False Discovery Rate threshold of 0.05. We conducted a gene enrichment analysis targeted for molecular function and cellular location using ClusterProfiler Package [47].

#### Targeted NAD+ metabolomics

Levels of NAD+ at treatment endpoints were measured by Ultra Performance Liquid Chromatography coupled with Mass Spectrometry as previously described [48] with some modifications. Under CO2 vapor, cell pellets were dissolved in chilled buffered methanol solution (75% methanol, 25% 10 mM HEPES pH 7.8) spiked with stable isotope-labeled internal standards normalization. Samples were then spun down at 16,000 x g for 10 minutes and transferred to new tubes. Pellets were saved for protein quantification as an additional normalization step. Supernatants were filtered using 4 mm 0.22 µM syringe filters (MilliporeSigma, Burlington, MA) and lyophilized overnight with a VirTis BT6KEL-85 freeze dryer (SP Scientific Warminster, PA). Lyophilized pellets were resuspended in a 50:50 acetonitrile/10 mM ammonium bicarbonate solution with 0.1% ammonium hydroxide. 5 µL of the extract was separated on a BEHAmide column (Waters, Milford MA) using an Acquity UPLC (Waters) and analyzed with a Xevo TQ (Waters) in multiple reaction monitoring mode (MRM). LC solvents were A: H2O with 10 mM ammonium acetate and 0.04% NH_4_OH. B: 95:5 Acetonitrile H_2_O with 10 mM ammonium acetate and 0.04% NH_4_OH for all metabolites. Gradient was: 5% A to 39% A at 6 minutes, to 56% A at 8 minutes, to 73% A at 8.2 minutes, and back to 5% A at 9 minutes. Each injection was 11 minutes long. Samples were run alongside an external standard curve for quantification.

#### Animals

##### MPTP-lesioning and tissue collection

C57BL/6 male mice, 8 to 10 weeks of age were purchased from Jackson Labs (Bar Harbor, Maine). 1-methyl-4- phenyl- 1,2,3,6-tetrahydropyridine (MPTP) (Selleck Chemicals Co., Houston, TX) was prepared to a concentration of 5 mg/kg free-base in 0.9% saline on the day of use. C57BL/6 mice were injected with 20 mg/kg free-base MPTP. 8 days after the last injection of MPTP, mice were killed by cervical dislocation and the brain quickly removed and placed on wet ice. Striatal tissues for HPLC analysis and western immunoblotting were collected fresh corresponding to anatomical regions from bregma 1.20 to bregma 0.60, with borders dorsal to the anterior commissure, ventral to the corpus callosum, medial to the lateral ventricle, and 2.5 mm lateral from midline, and frozen at -80 °C until analysis. All procedures utilizing MPTP lesioning and tissue collection were approved by the USC IACUC.

##### HPLC analysis of dopamine and its metabolites

Dopamine concentrations were determined according to an adaptation of Irwin et al. [49] from the method of Kilpatrick et al. [50]. Tissues for analysis were homogenized in 0.4 N perchloric acid and centrifuged at 12,000 X g to pellet precipitated protein and the supernatant used for HPLC analysis. The protein pellet was resuspended in 0.5 N NaOH and the total protein concentration determined using the BCA Protein Assay (Pierce, Rockford, IL), a Biotek Model Elx800 microplate reader (Biotek Instruments, Wincoski, VT) and KCjunior software. The concentrations of dopamine were assayed by HPLC with electrochemical detection. Samples were injected with an ESA (Chelmsford, MA) autosampler. Dopamine and its metabolites were separated by a 150 ×3.2 mm reverse phase 3-µm-diameter C-18 column (ESA) regulated at 28 °C. The mobile phase MD-TM (ESA) consisted of acetonitrile in phosphate buffer and an ion-pairing agent delivered at a rate of 0.6 ml/min. The electrochemical detector was an ESA model Coularray 5600 A with a four-channel analytical cell with three set potentials at -100, 50, and 220 mV. The HPLC was integrated with a Dell GX-280 computer with analytical programs including ESA Coularray for Windows software and the statistics package Prism 9 (GraphPad Software, San Diego, CA). Randomization (mixing the samples and order) of blinded samples during the process has been applied.

##### Western Immunoblotting

Striatal tissue samples were lysed in RIPA buffer with protease and phosphatase inhibitors (MSSAFE, Sigma), centrifuged at 16,000 x g and the soluble fraction collected for subsequent protein analysis. Total protein content was determined by BCA analysis (ThermoFisher Scientific) and 20 ug of protein resolved on a 10% Tris-Glycine gel by electrophoresis (BioRad, Hercules, CA). Total protein was transferred to nitrocellulose membranes (BioRad), blocked (Genesee Scientific) and probed with the following antibodies overnight at 4 °C: Tyrosine hydroxylase (MAB318, Millipore Scientific, Burlington, MA) and mouse anti-beta actin (1:5000, LI-COR, Cat# 926-42212, RRID: AB_2756372). Membranes were washed, incubated with corresponding goat anti-mouse or goat anti-rabbit conjugated near-infrared secondary fluorescent antibodies (1:5000, LI-COR, Cat# 926-32211, RRID: AB_621843; and Cat# 926-68070, RRID: AB_10956588), and scanned on an Odyssey imaging system (Licor, Lincoln, Nebraska, USA). Relative protein expression was quantified by optical density and normalized to beta-actin as loading control using the Licor imaging program.

##### Statistical analysis

The data is presented as the mean ± S.E.M. Significant differences were determined by unpaired Student’s t-tests and one-way ANOVA followed by Tukey’s post hoc test. Statistical analyses were performed by using the GraphPad Prism 8 software. Values of *<0.05, **<0.01, ***<0.001, ****<0.0001 were considered to be statistically significant.

## Results

### m.2158 T > C is associated with reduced risk of PD and with protective PD biomarkers

**Fig. 1:**
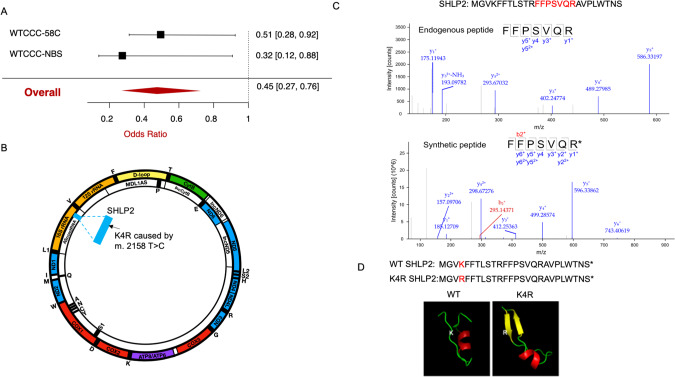
Identification of SHLP2 peptide in neuronal cells and demonstration of the importance of SHLP2 SNP in PD risk. **A** Forest plot for meta-analysis (pooled random effects) of m.2158 T > C SNP on the prevalence of PD (**B**) A schematic diagram of m.2158 T > C SNP and SHLP2 gene in a long noncoding RNA of the mitochondrial DNA. **C** Microprotein-targeted mass spectrometry identified SHLP2 in SH-SY5Y cells. (Top) MS2 scan of endogenous SHLP2 peptide from C8 enriched SH-SY5Y lysate ran in PRM mode (Bottom) MS2 scan of the spiked-in heavy labeled SHLP2 peptide ran in PRM mode. R* represents non-radioactive heavy isotope labeling at Arginine residue. **D** Amino acid sequence of WT and K4R SHLP2 and the predicted 3D model of peptides obtained via the program PEP-FOLD3.

Mitochondria-wide association studies (MiWAS) is similar to GWAS, but instead of focusing on nuclear genetic variations, MIWAS associates mitochondrial genetic variations with particular phenotypes. Although MiWAS approaches have identified mtSNPs that associated with various diseases, the underlying mechanisms are understudied. Based on the MiWAS analysis, we pose that mitochondrial genetic variation could alter mitochondrial microproteins [11, 18]. Here, we tested the hypothesis that mtSNPs within small open reading frames (sORFs) would associate with PD. Previously, genetic association studies from the Welcome Trust Case Control Consortium – 1958 Birth Cohort (2,197 PD cases were compared with 2,930 controls) and the UK National Blood Service (877 PD cases were compared with 2,729 controls) identified two mtDNA variants associated with the reduced risk of PD in Caucasians. Both m.2158 T > C (Fig. 1A; an odds ratio of 0.45) and m.11251 A > G reduce the risk of PD [6]. We then mapped these two significant mtSNPs to all potential mitochondrial sORFs. Whereas m.11251 A > G is not associated with any mitochondrial sORFs, m.2158 T > C replaces amino acid Lysine (K) with amino acid Arginine (R) in the 4th position of SHLP2. Thus, we focused on the role of m.2158 T > C on SHLP2 (Fig. 1B).

We examined whether m.2158 T > C in the SHLP2 sORF is associated with previously known PD risk factors. Specifically, we examined whether the m.2158 T > C is associated with plasma total cholesterol (TC), low-density lipoprotein cholesterol (LDL-C), and high-density lipoprotein cholesterol (HDL-C) because Ubiquinone-10 (coenzyme Q), a component of mitochondrial complex I, is a polyprenic molecule and is also part of the cholesterol biosynthetic pathway. Both mitochondrial complex I activity and cholesterol metabolism are significantly altered in patients with PD, and epidemiological studies showed both brain cholesterol and circulating cholesterol levels are associated with reduced PD risk and PD progression [51, 52]. To understand how the SHLP2 SNP is associated with known PD risk factors, we analyzed the association between the SHLP2 SNP and plasma cholesterol levels in control subjects (non-PD patients). We studied European ancestry individuals from three large population-based longitudinal cohorts: Health Retirement Study (HRS), Framingham Heart Study (FHS), and Cardiovascular Health Study (CHS). Based on the analysis, the alternative allele, m.2158 C is associated with higher TC (HRS: beta = 8.9, *p* = 0.025; FHS: beta =12.77, *p* = 0.01) and LDL-C (HRS: beta = 3.71, *p* = 0.43; FHS: beta = 9.37, *p* = 0.01) levels while no association was found with HDL-C (HRS: beta = 1.00, *p* = 0.48; FHS: beta = 0.37, *p* = 0.85) levels (Table 1, Supplementary Tables 1–4).

**Table 1:** Meta-analysis on m. 2158 T > C polymorphism and lipids.

	Effect	StdErr	*P* value	Direction
TC	8.14	2.85	0.004	++−
LDL	5.76	2.7	0.033	+++
HDL	0.39	1.06	0.715	++−

### Endogenous SHLP2 is detectable by mass spectrometry in a neuronal cell line

Endogenous SHLP2 was detected by the microprotein targeted proteomics approach, which enriched microproteins using a solid-phase extraction with C8 cartridges, followed by digestion with trypsin, and liquid chromatography-mass spectrometry [43]. First, we used synthetic light and heavy isotope-labeled SHLP2 peptides to develop a targeted parallel reaction monitoring assay. This analysis identified a detectable tryptic peptide, FFPSVQR, from SHLP2. Next, we analyzed lysates from SH-SY5Y cells with or without spiked the heavy isotope-labeled SHLP2 peptide and successfully identified endogenous SHLP2 expression (Fig. 1C). This data provides experimental evidence for SHLP2 translation from the non-coding RNA region of the mitochondrial DNA.

### Structural prediction of the K4R SHLP2

The analytical package PROVEAN (PROtein Variation Effect Analyzer; http://provean.jcvi.org) [53, 54] was used to predict the potential impact of the K4R substitution in SHLP2. The PROVEAN score for SHLP2 K4R replacement was -3.0, which is below the predicted cutoff score (=-2.5). Because the PROVEAN score is smaller than the given threshold, K4R SHLP2 is predicted to produce a functional change. Thus, we compared to the predicted structures of SHLP2 with K4R variant using the de novo modeling algorithm PEP-FOLD3 [55]. The predicted 3D structure of WT SHLP2 showed a loop-like structure with a small alpha- helix in the middle region, suggesting a flexible conformation. The K4R substitution potentially results in a significant alteration in the predicted 3D structure, forming an additional beta-sheet structure (Fig. 1D). Additionally, the change in the number of potential hydrogen bond from 3 in arginine to 2 in lysine could account for altered structure and function of the K4R SHLP2.

### Subcellular localization and stability of SHLP2 and K4R variant

**Fig. 2:**
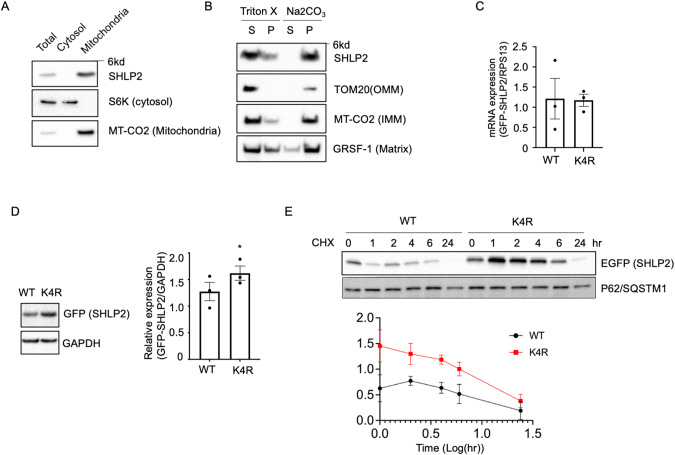
SHLP2 localization and stability. **A** Western blot of endogenous SHLP2 from subcellular fractions of HEK293 cells. **B** Extraction of endogenous SHLP2 from HEK293 mitochondria with buffer containing 1% Triton X-100 or Na_2_CO_3_ (C-E) HEK293 cells were transiently transfected with WT and K4R SHLP2 – tagged with EGFP. **C** mRNA expression of WT and K4R SHLP2 measured by qRT-PCR (**D**) (left) Representative image of total protein expression of WT and K4R SHLP2 (right) Quantification of the expression (**E**) Pulse-chase experiment of WT and K4R SHLP2. Cells were treated with 100ug/ml cycloheximide for the indicated time. p62/SQSTM1 is used as a loading control. Data are reported as mean ± SEM (n = 3/per group). Significant differences were determined by Student’s t-tests. *p < 0.05.

As protein localization in a cell is strongly associated with its function, we analyzed the putative subcellular localization of SHLP2 by using TargetP - 2.0 [56] and DeepLoc [57], a protein subcellular localization prediction algorithm based on deep learning. SHLP2 was predicted to be a mitochondrial membrane integrated protein. Indeed, with a custom antibody against full length SHLP2, SHLP2 is enriched in the mitochondria fraction, but not the cytoplasmic fraction (Fig. 2A). Sodium carbonate extraction is regarded as a canonical way to distinguish integral membrane proteins from other membrane‐associated proteins and soluble proteins. SHLP2 and other mitochondrial membrane proteins (TOM20 and MT-CO2) were resistant to sodium carbonate extraction suggesting SHLP2 is an integral membrane protein (Fig. 2B). The SHLP2 SNP causes an amino acid substitution of lysine with arginine in SHLP2. Lysine is a hotspot of post-translational modification, including ubiquitination, acetylation, and sumoylation. These post-translational modifications play essential roles in protein subcellular localization and/or stability. We thus examined the impact of the amino acid substation on protein stability. Creating an isogenic cell line using mtDNA editing could be an ideal approach to answer this question. Despite recent advances in mtDNA editing, the editing frequencies are still limited, up to 50%, and there is still off-target editing [58]. We thus overexpressed cells with plasmids carrying WT SHLP2 and K4R variant tagged with EGFP at the C-terminus and examined the stability of the peptides. HEK293 cells were transiently transfected with plasmids carrying either WT SHLP2 or K4R variant tagged with EGFP. Even though the mRNA expression of both WT and K4R SHLP2 was similar in HEK293 cells (Fig. 2C), the K4R SHLP2 showed higher fully expressed protein levels than WT (Fig. 2D). Furthermore, cycloheximide-treated pulse-chase experiments showed K4R SHLP2 is more stable than WT SHLP2 (Fig. 2E).

### SHLP2 localizes to mitochondrial complex 1

**Fig. 3:**
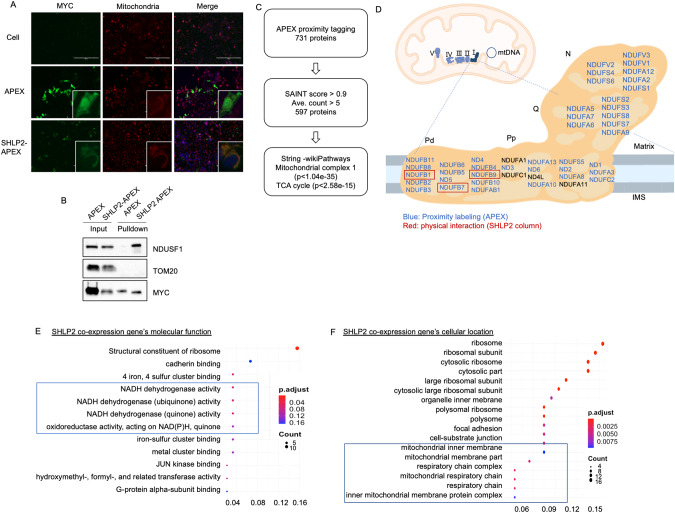
SHLP2 is localized in mitochondrial complex 1 and co-expressed with mitochondrial genes. **A** Mitochondrial localization of SHLP2-APEX. HEK293 cells were transfected with SHLP2-APEX or APEX control, fixed, and stained with anti-myc and anti-mitofilin antibodies to visualize APEX and mitochondria. Nuclei were stained with Hoechst. Scale bars are 200μm. **B** Representative western blot image of IMM and OMM proteins following SHLP2-APEX pulldown (n = 3). **C** Analysis of the SHLP2-APEX proximity labeling to remove false positives and perform pathway enrichment. **D** SHLP2 interacting proteins in the mitochondrial complex 1 (blue) SHLP2-APEX pull down (Red) SHLP2 peptide column. SHLP2 co-expression genes’ (**E**) molecular function and (**F**) cellular location in iPSC-derived dopaminergic neurons (iPSCs-derived dopaminergic neurons generated from 18 individuals). The color code represents the adjusted *p* value scale. The count is the number of genes significantly correlated for each term. The x axis is gene ratio, which is the ratio of input genes that are annotated in a term.

To understand the molecular function of SHLP2, we sought to identify SHLP2 interacting proteins. The identification of microprotein-protein interactions has proven to be a successful approach for the functional characterization of novel microproteins. However, traditional immunoprecipitation methods result in the enrichment of nonspecific interactions for microproteins. On the other hand, an in situ proximity tagging method that relies on an engineered ascorbate peroxidase 2 (APEX2) has been a useful tool to identify microprotein-protein interactions [45]. In this approach, APEX is fused to a protein of interest. Expression of the APEX fusion protein followed by treatment of cells with hydrogen peroxide in the presence of biotin-phenol covalently labels proteins proximal to the APEX fusion proteins with biotin. These biotinylated proteins can then be enriched and analyzed by mass spectrometry and western blotting, and because proteins adjacent to the APEX fusion protein are preferentially biotinylated, the resulting mass spectrometry data provide a readout of the protein environment around the fusion protein. Thus, we first utilized APEX2 proximity labeling to seek out SHLP2 interacting proteins. SHLP2-tagged-APEX showed mitochondrial localization, which supports the mitochondrial localization of SHLP2 (Fig. 3A). Based on our sodium carbonate extraction data, we hypothesized that SHLP2 is localized in either the inner mitochondrial membrane or the outer mitochondrial membrane. To test our hypothesis, we checked the biotinylation of one of the inner mitochondrial membrane proteins (NDUSF-1) and outer mitochondrial membrane proteins (Tom20) after treatment of SHLP2-APEX fusion-expressing HEK293 cells with biotin-phenol and H_2_O_2_. Western blotting of the biotin pull down revealed that NDUSF1 was biotinylated in cells transfected with the SHLP2-APEX fusion but not the APEX control (Fig. 3B). On the contrary, TOM20 was not biotinylated in cells transfected with both SHLP2-APEX and APEX control (Fig. 3B). These results suggest the localization of SHLP2 is in the inner mitochondrial membrane. We then analyzed the biotinylated proteins by mass spectrometry to capture proteins interacting with SHLP2 (Supplementary Table 5). We applied Significance Analysis of INTeractome Express (SAINT express), which uses a predicted distribution of spectral counts for a real protein-protein interaction to filter out false positives [59]. Application of SAINT to the SHLP2-APEX data set results in 597 proteins (Fig. 3C) with an average spectral count of greater than 5. Then, the enrichment analysis of proteins labeled by SHLP2-APEX using String-wikiPathways (v. 11.5) found proteins are enriched in mitochondrial complex 1 and TCA cycle (Fig. 3C). A previous mitochondrial matrix-targeted APEX study showed that mitochondrial matrix proteome from APEX labeling contain both soluble matrix proteins and inner mitochondrial membrane (IMM) proteins that contact the matrix space [60]. Likewise, mitochondrial membrane protein SHLP2-APEX appear to label both IMM proteins as well as mitochondrial matrix protein. In addition to APEX proximity labeling, we created a SHLP2 peptide column and a scrambled peptide column as a control to identify direct SHLP2 binding proteins (Supplemental Fig. 1A). This antibody-free approach helped to reduce the background interactions from antibody non-specificity while pulling down direct interacting partners of SHLP2. We added isolated mitochondrial protein extract and pulled down potential binding proteins. The elution was analyzed by mass spectrometry. We identified an enrichment of SHLP2 binding partner in mitochondrial complex 1 and TCA cycle proteins (Supplemental Fig. 1B). After analyzing data from APEX proximity labeling and the SHLP2 peptide column, we concluded that SHLP2 directly binds to the Pd modules of mitochondrial complex 1 (Fig. 3D). Mitochondrial complex 1 consists of four modules - N module (NADH oxidation), Q module (reduction of ubiquinone), Pd and Pp modules (proton pumping). These results suggest SHLP2 might play important roles in mitochondrial complex 1 related activity such as proton pumping.

To understand the molecular function of SHLP2 in neurons, we carried out a co-expression analysis on publicly available RNA-seq data derived from iPSCs-derived dopaminergic neurons [61]. Interestingly, significant genes that co-expressed with SHLP2 were related to NADH dehydrogenase activity (Fig. 3E) and the inner mitochondrial membrane (Fig. 3F); these associations align with our experimental data showing SHLP2 interaction with mitochondrial complex 1.

### SHLP2 protects against mitochondrial dysfunction in vitro

**Fig. 4:**
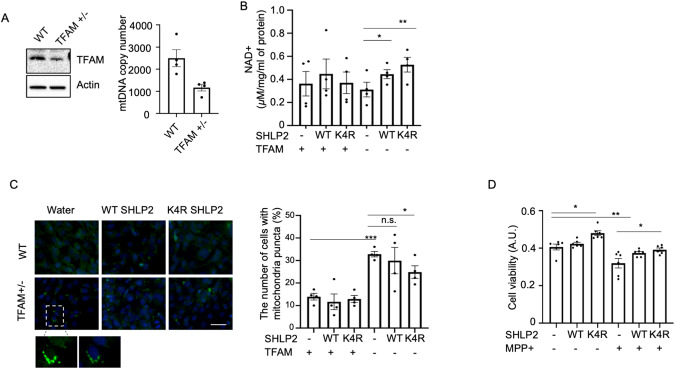
K4R SHLP2, produced by individuals who carry the m. 2158 T > C polymorphism, has superior protection against mitochondrial dysfunction. **A** TFAM expression and mtDNA copy number (**B**) NAD+ level was measured by mass spectrometry in WT and TFAM heterozygous knockout MEFs treated with 2.5μM WT and K4R SHLP2 for 24 hr. **C** (left) Immunostaining of mitochondria using COX IV antibodies. (right) The number of cells carrying mitochondria puncta was quantified. A total of 500-700 cells per group from four representative images were quantified. Scale bar is 50μm. **D** Cell viability was measured by MTT assay in SH-SY5Y cells treated with 2.5μM WT or K4R SHLP2 in the presence of 200μM MPP + . Data are reported as mean ± SEM (n = 4/per group). Significant differences were determined by Student’s t-tests. *p < 0.05, **p < 0.01, ***p < 0.001, n.s. = non significant.

Because we determined SHLP2 is in mitochondrial complex 1, we examined whether SHLP2 modifies mitochondrial function and prevents mitochondrial dysfunction. TFAM is a mitochondrial DNA binding protein essential for mitochondrial gene expression and genome maintenance. Patients with PD show reduced levels of TFAM and mtDNA copy number [62, 63]. Using TFAM heterozygous knockout mouse embryonic fibroblasts (MEFs), the potential protective role of SHLP2 against mitochondrial dysfunction was examined. Before we treated cells with synthetic SHLP2 peptide, we examined whether synthetic SHLP2 peptide enters to the mitochondria. First, MitoFates [64] predicted not only mitochondrial localization of SHLP2 but also a TOM20 recognition motif (amino acids 16-20) of SHLP2. Indeed, a SHLP2 column experiment for identification of SHLP2 binding proteins revealed that synthetic SHLP2 binds to TOM20 as well as other proteins in the mitochondrial protein import system. The results showed SHLP2 potentially binds to TOMM34, a chaperone to recruit proteins to transporters in outer mitochondrial membrane; SHLP2 binds to TOM20, then intermembrane space proteins (Timm9, Timm10, Timm10B), then is finally recruited to Tim50 in the inner mitochondrial membrane (Supplementary Fig. 2A). Moreover, rapid mitochondria immunoprecipitation showed SHLP2 was localized in the mitochondria after 30 min of treatment (Supplementary Fig. 2B). Based on these three approaches, we concluded that synthetic SHLP2 can be localized into the mitochondria and tested the hypothesis that synthetic SHLP2 peptides treatment protects against mitochondrial dysfunction found in TFAM heterozygous knockout MEFs. TFAM heterozygous knockout MEFs showed a 50% reduction in mtDNA copy number as reported elsewhere (Fig. 4A). Administration of WT SHLP2 and K4R SHLP2 did not alter levels of TFAM and Peroxisome proliferator-activated receptor-gamma coactivator (PGC-1 alpha) protein, the master regulator of mitochondrial biogenesis (Supplemental Fig. 3A–3C). Because mitochondrial complex 1 is involved in NADH oxidation, we examined the levels of NAD+ in TFAM heterozygous knockout MEFs. Exposure of MEFs cells to K4R SHLP2 resulted in increased NAD+ levels (Fig. 4B). Mitochondrial lysosome crosstalk has been recognized in previous studies via NAD + . TFAM knockout reduces NAD + , thereby including lysosomal dysfunction and aberrant mitochondrial morphology and increases mitochondrial mass due to the defective autophagosome-lysosome fusion [65]. Because K4R SHLP2 increased NAD+ levels in TFAM knockout cells, we examined the effect of K4R SHLP2 on mitochondrial morphology in the same cells. K4R SHLP2 significantly reduced the number of cells carrying enlarged mitochondria (Fig. 4C). Taken together, K4R SHLP2 can mitigate the mitochondrial dysfunction during TFAM reduction.

### SHLP2 has protection in SH-SY5Y cells treated with MPP + 

Reduced mitochondrial complex I activity has been found in PD pathology [66]. Mitochondrial complex 1 inhibitors such as MPTP and its metabolite MPP+ lead to neuronal cell death in vitro and in vivo, which is widely used for cellular and animal PD models [3, 67, 68]. Using SH-SY5Y cells treated with MPP + , the protective effect of WT SHLP2 and K4R variant in MPP + -induced cell death in vitro was examined. K4R SHLP2 treatment reduced the MPP + -induced cell death in SH-SY5Y cells (Fig. 4D).

### SHLP2 has protection in an MPTP mice model of PD

**Fig. 5:**
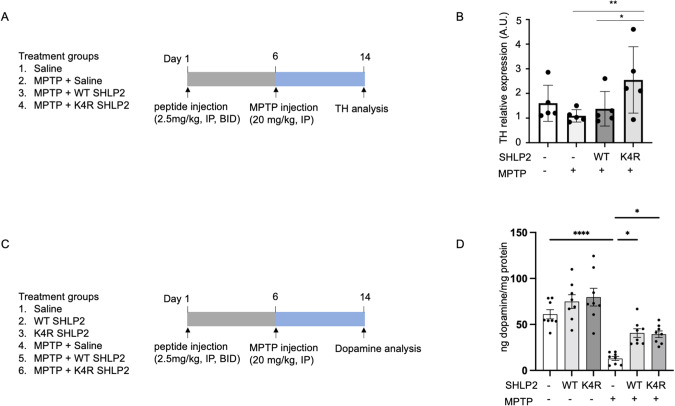
Pretreatment of mice with K4R SHLP2 shows protection in MPTP-lesioned mice. **A** Schematic diagram of treatment. **B** Tyrosine hydroxylase (TH) expression in the striatum from mice treated with or without MPTP and WT SHLP2 and K4R variant. (n = 5 per group). **C** Schematic diagram of treatment. **D** Dopamine contents were measured by using high-performance liquid chromatography with electrochemical detection in the striatum from mice treated with or without MPTP and WT and K4R SHLP2 (n = 8 per group). Data are reported as mean ± SEM. Significant differences were determined by one-way ANOVA followed by Tukey’s post hoc test *p < 0.05 ** < 0.01**** < 0.0001.

To examine the potential role of WT SHLP2 and K4R variant to provide protection against neurotoxin-induced dopamine-depletion in vivo, C57BL/6 mice exposed to MPTP were also administered WT SHLP2 or K4R variant starting 5 days prior to MPTP-induced cell death (Fig. 5A). Pretreatment of SHLP2 peptides could recapitulate the SHLP2 production in humans, which individuals carrying m.2158 T allele or m.2158 C allele produce WT SHLP2 or K4R variant, respectively. MPTP administration results in the degeneration of dopaminergic neurons within the substantia nigra, a brain region known for its projection to the striatum, leading to a consequential reduction in striatal dopamine levels. The assessment of dopaminergic neuron viability and functionality hinges on the quantification of tyrosine hydroxylase (TH) expression, a pivotal enzyme in dopamine biosynthesis [4]. To investigate the potential protective effects of SHLP2 against the loss of dopaminergic neurons triggered by MPTP, we performed western blot analysis to determine the TH expression levels in the striatum. Administration of K4R SHLP2 (2.5 mg/kg, IP, BID) protected against the loss of striatal TH expression in MPTP-treated mice while WT SHLP2 did not provide a similar degree of protection (Fig. 5B). Then, the dopamine levels in the striatum were measured by employing reverse-phase HPLC-electrochemical detection to determine whether SHLP2 can prevent the MPTP-induced reductions in dopamine (Fig. 5C). MPTP (20 mg/kg) induces a significant decrease in dopamine levels in the striatum of injected mice (Fig. 5D). WT and K4R SHLP2 administration rescues the MPTP-induced dopamine loss in the striatum compared to MPTP treated mice (Fig. 5D).

## Discussion

We determined that the m.2158 T > C polymorphism, which reduces PD risk, alters the MDP SHLP2 by mutating its fourth amino acid (K4R). We detected endogenous SHLP2 in neuronal cells using mass spectrometry. Through experimentation, we showed that SHLP2 microprotein was protective against mitochondrial dysfunction in vitro and in a mitochondrial toxin model of PD in mice.

Studies on the functional role of mtDNA polymorphisms have mainly focused on the 13 mtDNA-encoded respiratory chain proteins and free radical production [69, 70]. However, emerging studies have showed that small open reading frames within mtDNA also produces microproteins [9]. Here, we showed that PD protective SNP, m.2158 T > C affects a mitochondrial microprotein encoded from a long noncoding RNA of the mtDNA. m.2158 T > C changes the amino acid of the microprotein SHLP2 from lysine to arginine (K4R). We further demonstrated a protective role of K4R SHLP2 in in vitro and in vivo models of PD. We thus provided insights on the significance of the study investigating the functional role of mtSNPs on MDPs, which potentially determine the potential therapeutic targets. In addition, our work demonstrates how interpretation of previous associative studies with mtSNPs can be re-interpreted with a microprotein focus.

We identified SHLP2 by mass spectrometry in neuronal cells, which supports the coding potential of long noncoding RNA of the mtDNA. Traditional mass spectrometry often loses small proteins during sample preparation. Recent advancements in microprotein enrichment in mass spectrometry enabled us to identify the mitochondrial microprotein, SHLP2 in SH-SY5Y neuronal cells. Because peptidase causes microproteins to degrade rapidly, we began our sample preparation with a large quantity of starting materials. Next, we needed to detect microproteins by applying an enrichment. In previous research, four enrichment techniques have been proposed: acidic precipitation, 30 kDa MWCO filter, polyacrylamide gel electrophoresis, and reverse-phase (C8) column enrichment [43]. C8 column enrichment was originally developed to enrich plasma and tissue extracts for peptide hormones by removing larger molecular weight proteins before measurement by radioimmunoassay. Applying this method to enrich the lower molecular weight proteins gave excellent results to detect SHLP2 in SH-SY5Y neuronal cells.

We found SHLP2 is localized in mitochondrial complex 1, particularly the proton pumping module. K4R SHLP2 could bind to mitochondrial complex 1 more stably and prevent the decline of mitochondrial complex 1 activity (e.g., decline of NAD+ levels). Indeed, K4R SHLP2 treatment increased NAD+ levels in the TFAM heterozygous knockout MEFs. Given that declines in NAD+ have been shown to be harmful, we propose that an increased level of NAD+ by K4R SHLP2 could serve as a protective mechanism of action. A decline in NAD+ levels has been shown in PD patients, in vitro PD models, and in vivo PD models [71–74]. The blood of idiopathic PD patients revealed a decrease in the NAD + /NADH ratio [75]. The iPSC-derived neurons that express LRRK2 G2019S or mutant GBA1 showed mitochondrial dysfunction and a significant reduction of NAD+ levels [74]. Finally, a PD mouse model that was related to MPTP also showed reduction of NAD+ in the striatum and ventral midbrain but not in the frontal cortex [72]. In contrast, strategies to boost NAD+ metabolism, including the use of NAD+ precursors (e.g., NMN and NR) or small molecules that increase the rate of NAD+ biosynthesis or inhibit NAD+ degradation, have been used in current clinical trials [71]. Like NAD+ targeting therapies, we showed K4R SHLP2 not only increases NAD+ levels but also protected against MPTP mice model of PD.

A clinical study showed NAD+ precursors increase not only NAD+ levels but also total cholesterol and LDL [76]. Our meta-analysis showed that the PD-protective SHLP2 SNP is also associated with higher total cholesterol and LDL. Further studies examining NAD+ and its related metabolites levels in individuals carrying the SHLP2 SNP will confirm our observation in cells that SHLP2 increases NAD+ levels. These future studies could provide an insight on pharmacodynamics markers in development of PD therapeutics in the form of SHLP2 analogs. NAD+ was reduced in PD patients derived iPSCs carrying common mutations found in PD (LRRK2, GBA1) [73, 74]. The genetic interaction between SHLP2 SNP and PD mutations in the risk of PD and protective effects of K4R SHLP2 in PD patients-derived iPSCs carrying the PD mutations could be an essential direction to explore further in the future. Studies showed specific genetic mutations can manifest different responses to pharmacological therapies [77]. The future studies on the interaction between SHLP2 variant and PD mutations could be informative to develop SHLP2’s analog for personalized medicine.

In summary, we provide evidence that mitochondria microproteins play a central role in understanding disease-associated mtSNPs. We have taken a published mtSNP association that lacked a mechanism and have been able to demonstrate one possible reason for this association. We found that the mitochondrial inner membrane microprotein SHLP2 is affected by the m.2158 T > C polymorphism associated with reduced risk of PD. K4R SHLP2 produced by individuals carrying the SNP have higher cellular stability compared to WT SHLP2. Our data suggests that SHLP2 and its variants are PD protective microproteins and that administration of K4R may be able to protect against PD. Therefore, a deeper understanding of the effects of this genetic polymorphism and its related mitochondrial microprotein will provide a basis for developing novel therapeutic targets in PD.

### Supplementary information

Supplemental figure

Supplementary table 1

Supplementary table 2

Supplementary table 3

Supplementary table 4

Supplementary table 5

Supplementary table 6

## Data Availability

The SHLP2 mass spectrometry proteomics data have been deposited to the ProteomeXchange Consortium via MassIVE (http://proteomecentral.proteomexchange.org) via the PRIDE partner repository with the data set identifier PXD034482. The APEX and SHLP2 column mass spectrometric raw data are deposited at ftp://MSV000089658@massive.ucsd.edu (password: Shlp2_2022) with the MassIVE ID MSV000087928. (after publication: ftp://massive.ucsd.edu/MSV000089658/).
