# Supplementary material for: A naturally occurring variant of SHLP2 is a protective factor in Parkinson’s disease
Source: Mol Psychiatry. 2024 Jan 3;29(2):505–17. doi: 10.1038/s41380-023-02344-0 (PMC11116102; doi:10.1038/s41380-023-02344-0)
Supplement: Supplementary file 1 — Supplemental figure [file 41380_2023_2344_MOESM1_ESM.pdf]

Supplementary figure 1. SHLP2 column mass spectrometry revealed mitochondrial complex 1 protein bound to SHLP2 peptide
(A) Schematic diagram of SHLP2 column mass spectrometry analysis
(B) Analysis of the SHLP2 column pulldown-MS to remove false positives and background proteins

Supplemental figure 2. Synthetic SHLP2 localized in the mitochondria (A) A schematic of mitochondrial protein import system. SHLP binding proteins from a SHLP2 column mass spectrometry experiment were highlighted in red (B, top) rapid mitochondrial isolation method using immunoprecipitation (B, bottom) western blot of SHLP2 and subcellular compartments' markers.

Supplementary figure 3. SHLP2 does not change TFAM and PGC-1alpha expression in TFAM +/- MEFs
(A) Western blot of TFAM and PGC-1alpha
(B) Quantification of TFAM expression
(C) Quantification of PGC-1alpha expression
Data are reported as mean ± SEM (n=3/per group). Significant differences were determined by Student's t-tests. $^*p<0.05$

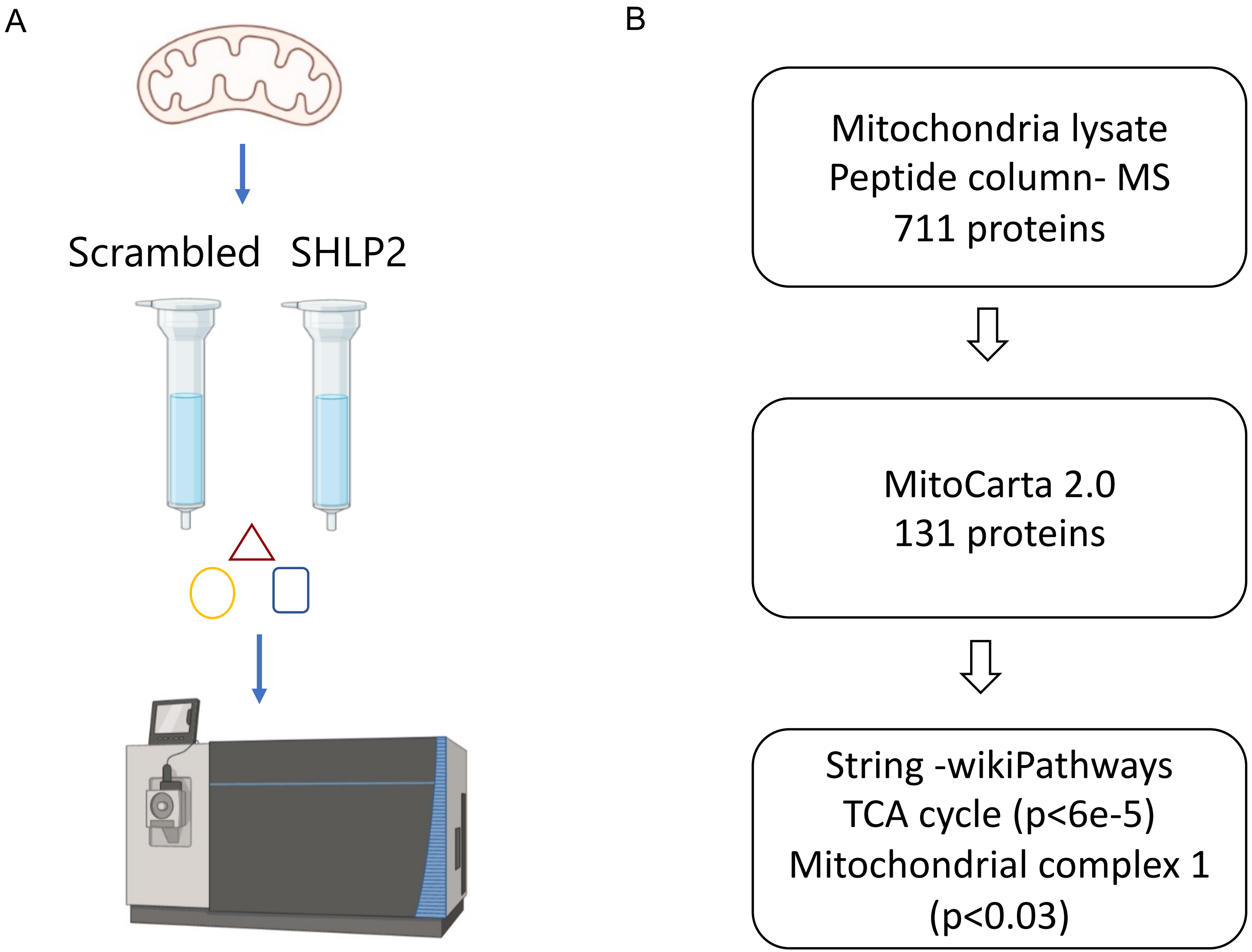
A
Scrambled
SHLP2
B
Mitochondria lysate
Peptide column- MS
711 proteins
MitoCarta 2.0
131 proteins
String -wikiPathways
TCA cycle (p<6e-5)
Mitochondrial complex 1
(p<0.03)

A

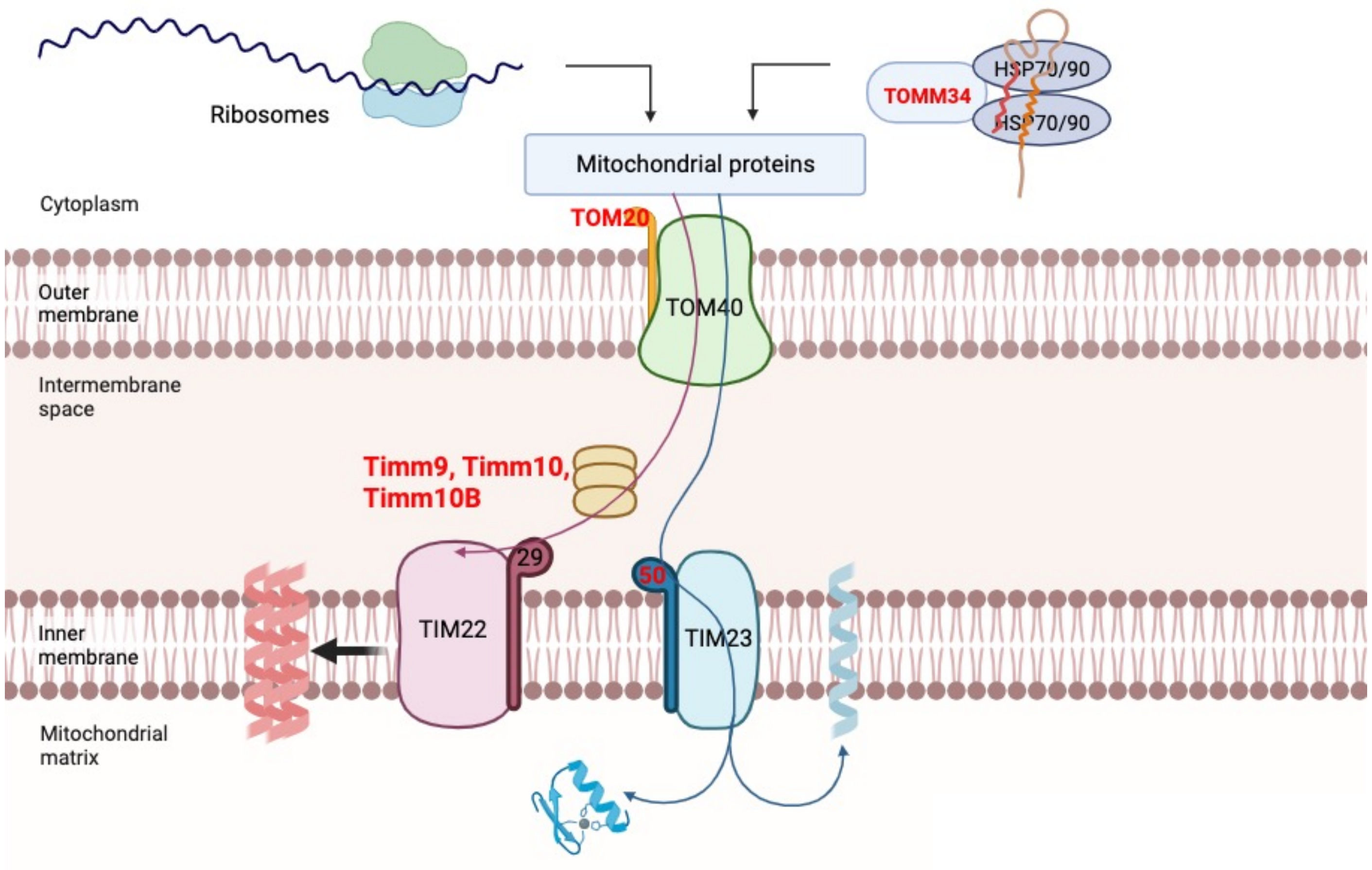

Ribosomes
Mitochondrial proteins
TOMM34
HSP70/90
HSP70/90
Cytoplasm
TOM20
TOM40
Outer membrane
Intermembrane space
Timm9, Timm10, Timm10B
29
50
TIM22
TIM23
Inner membrane
Mitochondrial matrix

B

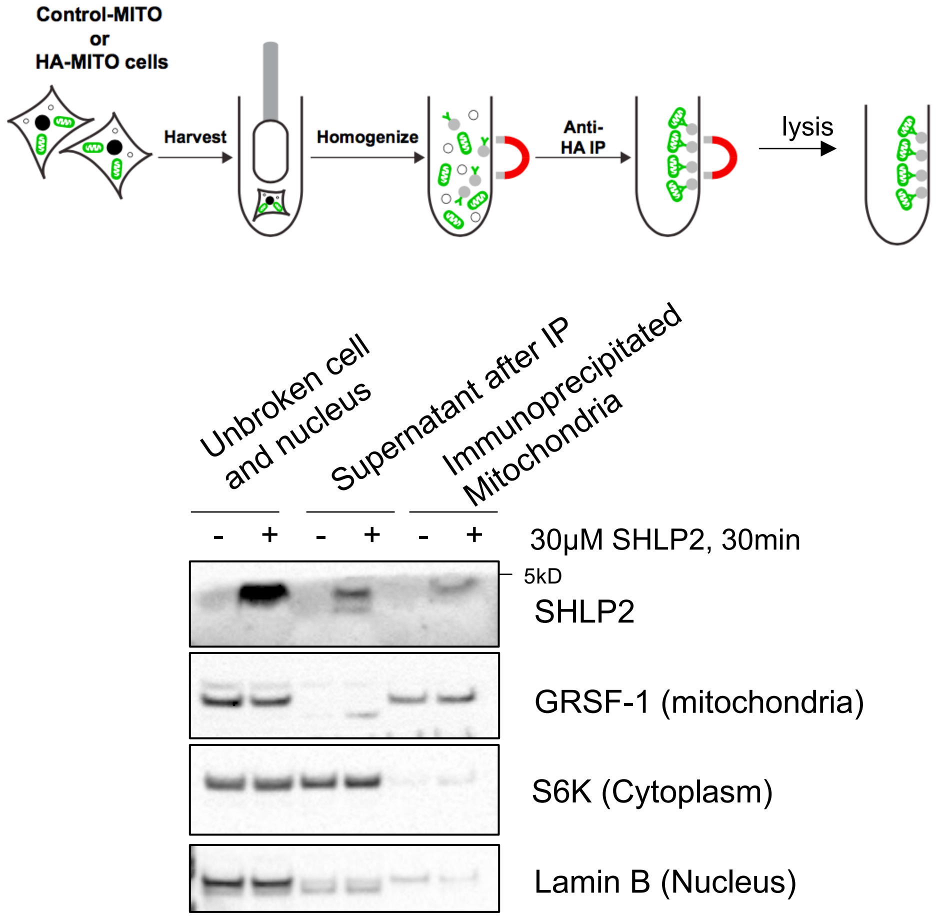

Control-MITO or HA-MITO cells
Harvest
Homogenize
Anti-HA IP
lysis
Unbroken cell and nucleus
Supernatant after IP
Immunoprecipitated Mitochondria
- + - + - +
30μM SHLP2, 30min
5kD
SHLP2
GRSF-1 (mitochondria)
S6K (Cytoplasm)
Lamin B (Nucleus)

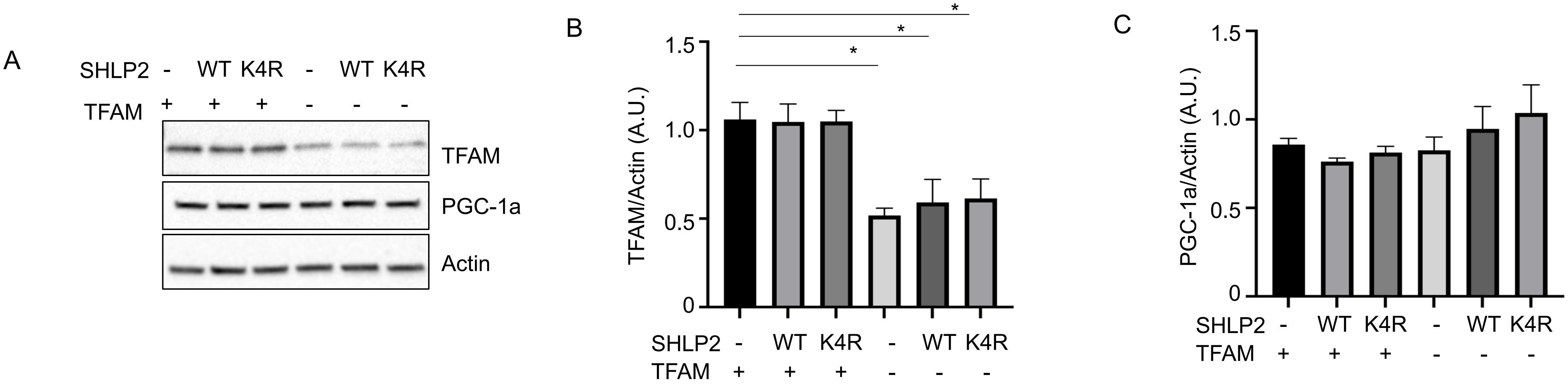
A
SHLP2 - WT K4R - WT K4R
TFAM + + + - - -
TFAM
PGC-1a
Actin
B
TFAM/Actin (A.U.)
1.5
1.0
0.5
0
*
*
*
SHLP2 - WT K4R - WT K4R
TFAM + + + - - -
C
PGC-1a/Actin (A.U.)
1.5
1.0
0.5
0
SHLP2 - WT K4R - WT K4R
TFAM + + + - - -
